# Electrophysiological effects of adipose graft transposition procedure (AGTP) on the post-myocardial infarction scar: A multimodal characterization of arrhythmogenic substrate

**DOI:** 10.3389/fcvm.2022.983001

**Published:** 2022-09-20

**Authors:** Raquel Adeliño, Daina Martínez-Falguera, Carolina Curiel, Albert Teis, Roger Marsal, Oriol Rodríguez-Leor, Cristina Prat-Vidal, Edgar Fadeuilhe, Júlia Aranyó, Elena Revuelta-López, Axel Sarrias, Víctor Bazan, Joan F. Andrés-Cordón, Santiago Roura, Roger Villuendas, Josep Lupón, Antoni Bayes-Genis, Carolina Gálvez-Montón, Felipe Bisbal

**Affiliations:** ^1^ICREC Research Program, Germans Trias i Pujol Research Institute (IGTP), Barcelona, Spain; ^2^Faculty of Medicine, University of Barcelona, Barcelona, Spain; ^3^Boston Scientific Department, Barcelona Delegation, Barcelona, Spain; ^4^Heart Institute (iCOR), Germans Trias i Pujol University Hospital, Barcelona, Spain; ^5^CIBER Cardiovascular, Instituto de Salud Carlos III, Madrid, Spain; ^6^Faculty of Medicine, University of Vic-Central University of Catalonia (UVic-UCC), Vic, Spain; ^7^Department of Medicine, Can Ruti Campus, Autonomous University of Barcelona, Barcelona, Spain

**Keywords:** myocardial infarction, adipose graft transposition procedure, myocardial repair, arrhythmic risk, ventricular tachycardia, mapping

## Abstract

**Objective:**

To assess the arrhythmic safety profile of the adipose graft transposition procedure (AGTP) and its electrophysiological effects on post-myocardial infarction (MI) scar.

**Background:**

Myocardial repair is a promising treatment for patients with MI. The AGTP is a cardiac reparative therapy that reduces infarct size and improves cardiac function. The impact of AGTP on arrhythmogenesis has not been addressed.

**Methods:**

MI was induced in 20 swine. Contrast-enhanced magnetic resonance (ce-MRI), electrophysiological study (EPS), and left-ventricular endocardial high-density mapping were performed 15 days post-MI. Animals were randomized 1:1 to AGTP or sham-surgery group and monitored with ECG-Holter. Repeat EPS, endocardial mapping, and ce-MRI were performed 30 days post-intervention. Myocardial SERCA2, Connexin-43 (Cx43), Ryanodine receptor-2 (RyR2), and cardiac troponin-I (cTnI) gene and protein expression were evaluated.

**Results:**

The AGTP group showed a significant reduction of the total infarct scar, border zone and dense scar mass by ce-MRI (*p* = 0.04), and a decreased total scar and border zone area in bipolar voltage mapping (*p* < 0.001). AGTP treatment significantly reduced the area of very-slow conduction velocity (<0.2 m/s) (*p* = 0.002), the number of deceleration zones (*p* = 0.029), and the area of fractionated electrograms (*p* = 0.005). No differences were detected in number of induced or spontaneous ventricular arrhythmias at EPS and Holter-monitoring. SERCA2, Cx43, and RyR2 gene expression were decreased in the infarct core of AGTP-treated animals (*p* = 0.021, *p* = 0.018, *p* = 0.051, respectively).

**Conclusion:**

AGTP is a safe reparative therapy in terms of arrhythmic risk and provides additional protective effect against adverse electrophysiological remodeling in ischemic heart disease.

## Introduction

Despite rapid progress over the past two decades, myocardial infarction (MI) remains a leading cause of disability and mortality. Myocardial revascularization strategies have significantly improved the natural history of MI, yet post-infarct scar continues to produce ventricular dysfunction, heart failure, and sudden death as a result of ventricular arrhythmias (1, 2). Development of new reparative therapies remains a priority and continues to be a challenge in the field of biomedical research.

The adipose graft transposition procedure (AGTP) is a reparative therapy based on placing an autologous pericardial adipose pedicle over the infarct scar. The adipose flap contains mesenchymal stromal cells with immunomodulatory and angiogenic properties capable of migration into the infarcted myocardium. Since the pedicle maintains its own vascularization, AGTP ensures the viability of the mesenchymal stromal cells over time, thereby facilitating their reparative effect (3–5).

We and others have shown that AGTP therapy reduces the size of the scar and increases vascularization in the infarcted area in a porcine model of chronic MI, with absence of arrhythmic events at 30 days post-intervention (4). In the first-in-human phase I trial, this therapy proved to be safe in terms of arrhythmic events, hospital admissions, and mortality (5). Currently, AGTP efficacy is being tested in a multicenter, randomized, controlled phase II-III clinical trial (AGTP II Trial, NCT02798276) (6).

Despite the potential beneficial effects of AGTP and other cardiac reparative therapies, a thorough assessment of their arrhythmogenic profile remains mandatory. Both pro- and antiarrhythmic mechanisms have been attributed to cell therapies, in most cases through *ex vivo* studies (7–13).

During the pre-clinical and clinical evaluation of AGTP therapy, no arrhythmic events have been detected, although they were never actively sought and their true incidence could have been underestimated. Accordingly, the objective of the study was to characterize the effect of AGTP therapy on tissue characteristics and the electrophysiological properties of ischemic scar in a porcine model of chronic MI.

## Materials and methods

This study was approved by the Animal Experimentation Unit Ethics Committee of the Germans Trias i Pujol Health Research Institute (IGTP) and by government authorities (Generalitat de Catalunya; Code: 10558). It complied with all guidelines concerning the use of animals in research and teaching as defined by the Guide for the Care and Use of Laboratory Animals (14).

### Study design

**FIGURE 1:**
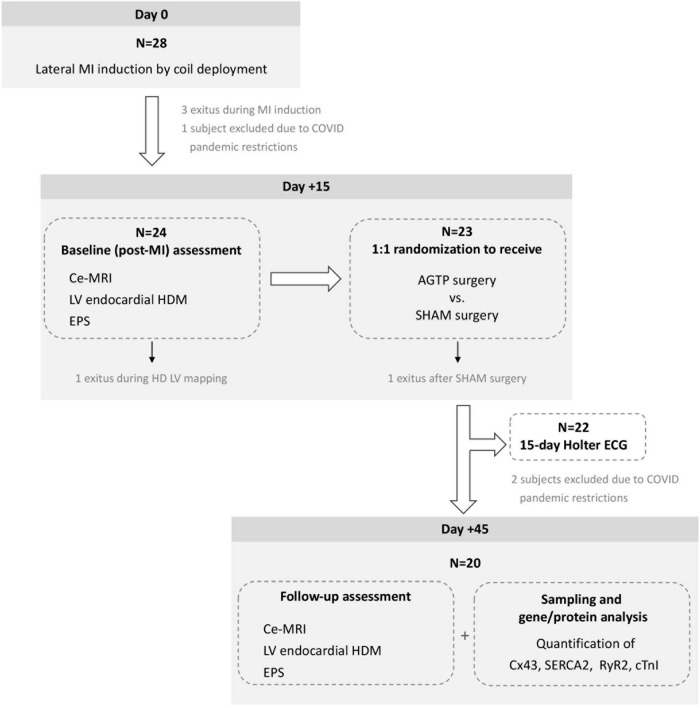
Chronogram and workflow of the study. ce-MRI, contrast-enhanced magnetic resonance imaging; HD, high density; LV, left ventricular; EPS, electrophysiological study.

The study design is depicted in Figure 1. Twenty-eight crossbreed Landrace X Large White pigs (50% females) underwent MI induction. At 15 days post-MI, evaluation with contrast-enhanced magnetic resonance imaging (ce-MRI), electrophysiological study (EPS), and left ventricular (LV) endocardial high-density mapping (HDM) were performed. Animals were randomly assigned to AGTP or sham surgery (Sham group). Follow-up evaluation (ce-MRI, EPS, HDM) and gene/protein analysis were performed 45 days post-MI (30 days post-surgery). All procedures were carried out under general anesthesia and endotracheal intubation (extended details can be found in Supplementary material).

### Myocardial infarction swine model

The model of non-reperfused MI was previously described (15). Briefly, a left lateral MI was induced by percutaneous deployment of 1–3 coils (VortX-18 Diamond 3 mm/3.3 mm coil, Boston Scientific/Target, Natick, MA, United States) at the proximal first marginal branch of the circumflex artery or another branch as needed (4, 15). Coronary occlusion (TIMI flow score = 0) was confirmed by angiography in all cases. To assess differences in myocardial damage between groups, cardiac troponin-I (cTnI) serum levels were analyzed in blood samples (ARCHITECT STAT High Sensitive Troponin-I; Architect i2000) collected at baseline and 2 h post-MI.

### Adipose graft transposition procedure

Two weeks after MI induction, pigs underwent left lateral thoracotomy in the fourth intercostal space to expose the heart protected by the pericardium. The pericardial adipose tissue was detached from the fibrous layer of the pericardium, maintaining its vascularization to create the adipose flap. After pericardiectomy, myocardial scar was localized in the LV lateral wall and covered by the vascularized adipose graft in AGTP-treated pigs and sealed with cyanoacrylic-based surgical glue (Glubran^®^2, Cardiolink, Barcelona, Spain), as previously described (3, 4). In the Sham group, the pericardium and pericardial adipose tissue were removed.

### Magnetic resonance imaging acquisition and post-processing

Ce-MRI was performed by 3T MRI scanner (Canon Vantage Galan; Ota, Tokyo, Japan), using a phased-array four-channel surface coil and ECG gating. All animals were under general anesthesia and endotracheal intubation. All images were acquired during repeated ventilator apneas and synchronized with the ECG. Fast field echo (FFE) cine images were acquired in 2, 3, and 4 chamber views and in short-axis orientation covering left ventricle from base to apex. Slice thickness was 8 mm and no gap was used between short-axis slices. A set of conventional delayed enhancement images was obtained 10 min after bolus injection of gadolinium-derived contrast agent (Gadobutrol 0.2 mmol/kg, Gadovist^®^, Bayer, Berlin, Germany) using a well-established segmented 2D inversion recovery gradient-echo sequence. Additionally, a whole-heart high spatial resolution delayed enhancement was acquired using a free-breathing, navigator-gated 3D inversion-recovery gradient-echo technique.

Cardiac structure and function were analyzed with Medis Medical Imaging^®^ (Leiden, Netherlands) and included end-diastolic volume (EDV), end-systolic volume (ESV), LV ejection fraction (LVEF), and LV myocardial mass (16).

Scar characterization with late gadolinium enhancement sequences was post-processed with the ADAS 3D^®^ software (Galgo Medical, Barcelona, Spain) and included quantification of scar subtypes (17–21). Ce-MRI provides the capability to assess scar 3-dimensionality by means of full-thickness analysis. Thresholds of > 60, 40–60%, and < 40% of maximum pixel intensity were used to identify dense scar, heterogeneous border zone (BZ) tissue, and normal tissue, respectively. The number and mass of MRI-detected BZ corridors were quantified. BZ corridors are automatically detected as corridors of BZ tissue between two core areas or between a core area and a valve annulus and have been correlated with slow conducting channels in sinus rhythm and ventricular tachycardia (VT) isthmuses (19, 21).

### Electrophysiological study and endocardial mapping

LV endocardial HDM was performed via retrograde aortic access by means of the 64-pole basket catheter (Intellamap Orion, Boston Scientific, Massachusetts, United States) and Rhythmia HDx 3D mapping system (Boston Scientific, Massachusetts, United States) during right ventricular pacing at a fixed cycle length of 400–450 ms, depending on baseline heart rate (same cycle length was used in baseline and follow-up maps). Programmed ventricular stimulation protocol was performed with S1 trains (600–500–400 ms) and up to S5 (ventricular refractory period or 170 ms). Mapping of induced VT was attempted whenever hemodynamic conditions allowed. Extended details regarding signal filtering and acquisition can be found in Supplementary material.

Voltage and activation maps were analyzed off-line with Rhythmia and self-customized Paraview-based software.^1^ Conventional cut-offs were used for bipolar (0.5–1.5 mV) and unipolar (6.7 mV) (22, 23) voltage. Conduction velocity (CV) was determined for every pair of contiguous points. Areas of CV were quantified for every 0.2 m/s steps (< 0.2–4 m/s). Lumipoint module was used to determine areas of post-QRS activation and slow conduction zones, including number of deceleration zones (DZ, defined as ≥ 3 isochrones within 1 cm out of 8 isochrones comprising the entire ventricular activation window) (24), and areas of complex fractionated electrograms (≥ 4 deflections of the bipolar electrogram) (25, 26).

### Heart rhythm monitoring

Half of the animals in each group were monitored with 3-lead ECG-Holter (AFT-1000-A Holter Supplies, Pairs, France) for 15 days post-surgery. Baseline rhythm, number and length of sustained and non-sustained VT episodes, and the burden of premature ventricular contractions (PVC) were quantified.

### Gene and protein expression analysis

Expression levels of Connexin 43 (*Cx43)*, cardiac Troponin-I (*cTnI)*, sarco/endoplasmic reticulum Ca^2+^-ATPase (*SERCA2)*, and Ryanodine receptor-2 (*RyR2)* genes were measured by quantitative real-time PCR (qRT-PCR) (Supplementary Table 1) from infarct core, BZ, and remote myocardium in a subset of animals (Sham *n* = 7; AGTP *n* = 6).

For western blot analysis, 50 μg of protein from the infarct core was used to determine cTnI, Cx43, SERCA2, RyR2, and α-tubulin protein expression. Immunohistochemical analyses were performed with anti-Cx43, SERCA2, α-SMA, and cTnI antibodies. Further details about tissue collection, gene and protein expression, and immunohistochemical analysis are included in Supplementary material.

### Statistical analysis

Continuous data were reported as mean ± SD for normal-distributed variables or median ± IQR for non-normal distributed variables. Categorical data were presented as percentages. Differences between groups in continuous variables were compared using Student’s *t*-test, ANOVA Greenhouse-Geisser correction, or Wilcoxon-rank test when the variable was not normally distributed. Chi-Square and Fisher Exact tests were used to compare categorical variables. Values of *p* < 0.05 were considered significant. Analyses were performed with Stata software (version 12, StataCorp, College Station, TX) and SPSS (19.0.1 version, SPSS, Inc., Chicago, IL).

## Results

### Study population

Initially, 28 pigs were enrolled between September 2019 and January 2021. Due to COVID-19 pandemic restrictions, the experiment was temporarily suspended and 3 animals had to be excluded because of lockdown. Five animals died before completion of the study: 3 during MI induction [due to ventricular fibrillation (VF), atrioventricular block, and coronary dissection, respectively], 1 during baseline mapping (due to VF), and 1 following sham surgery (due to VF). The remaining 20 subjects (33.9 ± 3.2 kg) were allocated 1:1 to receive AGTP (*n* = 10; 50% female) or sham surgery (*n* = 10; 50% female). All completed the study interventions and follow-up (Figure 1). Baseline characteristics are detailed in Table 1.

**TABLE 1:** Baseline characteristics of AGTP and sham-surgery animals.

	AGTP	Sham	*P*
**Morphometric data**			
Sex (female: N,%)	5, 50%	5, 50%	1.000
Weight (Kg, mean ± SD)	34.7 ± 3.1	34.1 ± 3.9	0.686
**Characteristics of substrate assessed by electroanatomical mapping**			
Total scar area (cm^2^, mean ± SD)	8.4 ± 5.2	5.4 ± 4.1	0.172
BZ area (cm^2^, mean ± SD)	5.6 ± 4.4	3.8 ± 2.6	0.100
Dense scar area (cm^2^, median ± IQR)	1.5 ± 2.3	0.5 ± 2.6	0.596
Unipolar voltage area < 6.7 V (mm^2^, median ± IQR)	80.1 ± 550.5	155.3 ± 187.8	0.677
Area of velocity < 0.2 m/s (mm^2^, median ± IQR)	5.3 ± 8.6	0.3 ± 2.3	0.012
Area of velocity < 0.4 m/s (mm^2^, median ± IQR)	6.4 ± 9.8	1.9 ± 7.7	0.080
Number of DZ (N, mean ± SD)	1.6 ± 1.3	0.8 ± 0.8	0.110
Post-QRS activation area (cm^2^, median ± IQR)	2.0 ± 1.5	1.1 ± 1.3	0.168
Area of electrograms with ≥ 4 deflections (cm^2^, mean ± SD)	4.1 ± 1.9	2.8 ± 1.2	0.120
**Characteristics of substrate assessed by MRI**			
iLVEDV (ml, mean ± SD)	124.2 ± 17.6	114.2 ± 19.2	0.271
iLVESV (ml, mean ± SD)	79.0 ± 17.6	71.9 ± 21.0	0.403
LVEF (%, mean ± SD)	38.0 ± 11.2	35.6 ± 12.0	0.674
Percentual scar size (%, mean ± SD)	10.0 ± 2.8	9.0 ± 6.1	0.700
Total scar mass (g, mean ± SD)	7.9 ± 2.9	5.8 ± 3.3	0.165
BZ mass (g, mean ± SD)	4.9 ± 1.8	4.0 ± 2.7	0.446
Dense scar mass (g, mean ± SD)	1.8 ± 1.0	3.1 ± 1.7	0.060
Corridors, number (N, median ± IQR)	1.0 ± 1.0	0.5 ± 1.0	0.241
Corridors, mass (g, mean ± SD)	0.5 ± 0.3	0.3 ± 0.3	0.074

BZ, border zone; DZ, deceleration zones; iLVEDV, indexed left ventricle end-diastolic volume; iLVESV, indexed left ventricle end-systolic volume; LVEF, left ventricular ejection fraction.

### Myocardial infarction assessment

No significant differences in the increase of circulating levels of cTnI after MI were found between AGTP and sham-surgery animals, indicating similar magnitude of myocardial ischemic damage between groups (*p* = 0.286).

### Magnetic resonance imaging characterization of myocardial infarction

**FIGURE 2:**
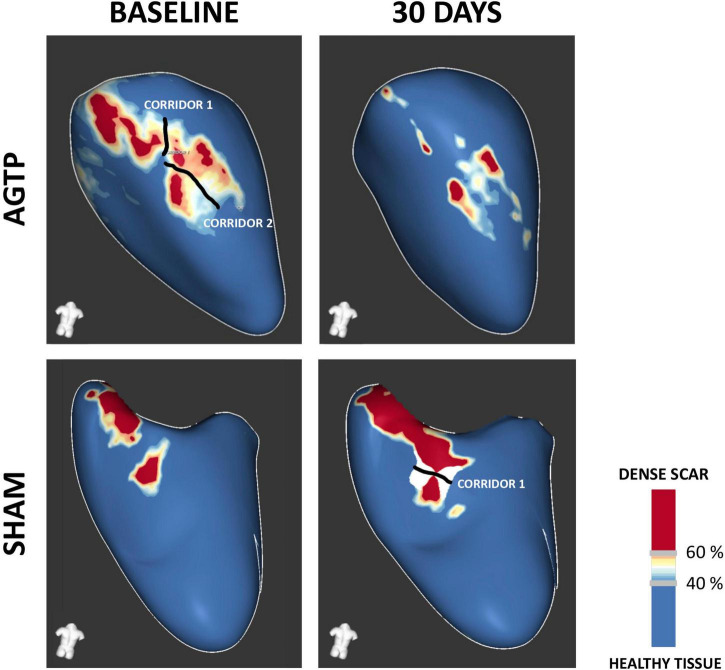
Contrast-enhanced MRI-based tissue characterization. Representative examples of post-MI substrate remodeling at 30-day follow-up. Favorable remodeling was observed in the AGTP group **(upper panel)**, with a significant reduction in border zone (BZ) and dense scar and a trend toward reduced BZ corridors. Sham group **(lower panel)** showed increased scar volume and a trend toward development of new BZ corridors.

Baseline iLVEDV, iLVESV, LVEF, total scar mass, BZ mass, dense scar mass, and number of corridors were similar between groups (Table 1). Thirty days after treatment, scar analysis by ce-MRI showed significant reduction in total scar mass (–2.2 ± 2.5 g vs. + 1.7 ± 3.2 g, *p* = 0.012), BZ mass (–1.6 ± 1.6 g vs. + 1.1 ± 2.5 g, *p* = 0.014), and dense scar mass (–0.6 ± 1.1 g vs. + 0.5 ± 1.1 g, *p* = 0.042) in the AGTP group compared to the Sham group. A trend toward reduction in the number and mass of VT corridors was observed (–0.4 ± 1.3 vs. + 0.4 ± 1.2, *p* = 0.2 and –0.2 ± 0.5 g vs. + 0.1 ± 0.4 g, *p* = 0.14, respectively) (Figure 2). There were no differences between groups regarding LVEF or LV volumes (Table 2). Intragroup variation analysis is included in Supplementary Table 2.

**TABLE 2:** Results of MRI, LV endocardial HDM and rhythm monitoring of AGTP and sham-surgery animals at 30-day follow-up from the surgery.

	AGTP	Sham	*P*
**MRI LV and scar analysis**			
iLVEDV (ml, mean ± SD)	–3.2 ± 18.5	+ 2.4 ± 18.9	0.532
iLVESV (ml, mean ± SD)	–5.7 ± 18	–4.2 ± 16	0.938
LVEF (%, mean ± SD)	+ 2.6 ± 9.4	+4.4 ± 9.6	0.695
Total scar mass (g, mean ± SD)	–2.2 ± 2.5	+ 1.7 ± 3.2	0.012
BZ mass (g, mean ± SD)	–1.6 ± 1.6	+ 1.1 ± 2.5	0.014
Dense scar mass (g, mean ± SD)	–0.6 ± 1.1	+ 0.5 ± 1.1	0.042
Corridors, number (g, mean ± SD)	–0.4 ± 1.3	+ 0.4 ± 1.2	0.203
Corridors, mass (g, mean ± SD)	–0.2 ± 0.5	+ 0.1 ± 0.4	0.136
**HD electroanatomical mapping**			
Total scar area (cm^2^, mean ± SD)	–2.2 ± 3.4	1.1 ± 0.5	0.012
BZ area (cm^2^, median ± IQR)	–1.9 ± 2.4	+ 1.0 ± 1.9	< 0.001
Dense scar area (cm^2^, mean ± SD)	0.3 ± 1.3	–0.2 ± 0.9	0.353
Unipolar low voltage area (mm^2^, median ± IQR)	+ 0.1 ± 2.8	–0.2 ± 7.9	0.449
Area of velocity < 0.2 m/s (mm^2^, mean ± SD)	–3.9 ± 3.8	+ 1.6 ± 3.1	0.002
Area of velocity < 0.4 m/s (mm^2^, mean ± SD)	–3.1 ± 10.3	1.0 ± 6.7	0.087
DZ (N, mean ± SD)	–0.3 ± 0.9	+ 0.8 ± 0.9	0.029
Post-QRS activation area (cm^2^, median ± IQR)	–0.4 ± 1.5	+ 0.8 ± 2.8	0.069
Area of electrograms with ≥ 4 deflections (cm^2^, mean ± SD)	–1.1 ± 1.3	+ 0.6 ± 0.9	0.005
**Rhythm monitoring**			
Number of PVC (mean ± SD)	500 ± 472	600 ± 442	0.687
Number of non-sustained VT episodes (mean ± SD)	6.0 ± 8.6	4.5 ± 4.9	0.572
Total time in VT (seconds, mean ± SD)	13.0 ± 25.3	8.2 ± 9.6	0.345

BZ, border zone; DZ, deceleration zones; iLVEDV, indexed left ventricle end-diastolic volume; iLVESV, indexed left ventricle end-systolic volume; LVEF, left ventricular ejection fraction; PVC, premature ventricular contractions; VT, ventricular tachycardia.

### Endocardial mapping and electrophysiological study

**FIGURE 3:**
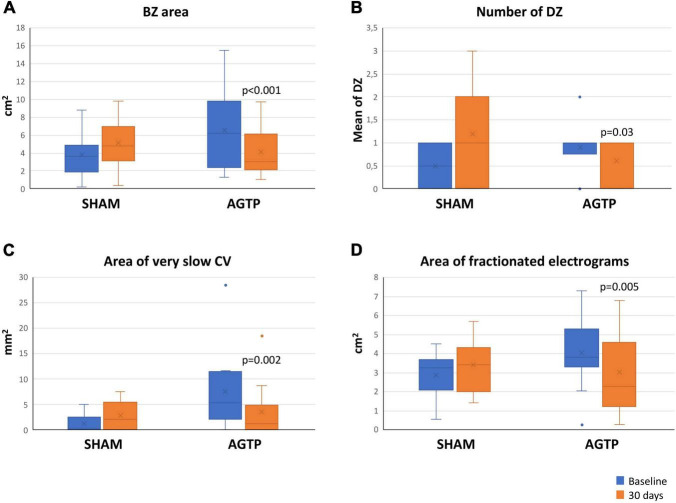
Baseline and follow-up mapping data. Boxplots shows significant differences in the area of border zone tissue **(A)**, number of deceleration zones **(B)**, very-slow conduction velocity **(C)**, and highly fractionated electrogram area **(D)**. BZ, border zone; CV, conduction velocity; DZ, deceleration zones.

**FIGURE 4:**
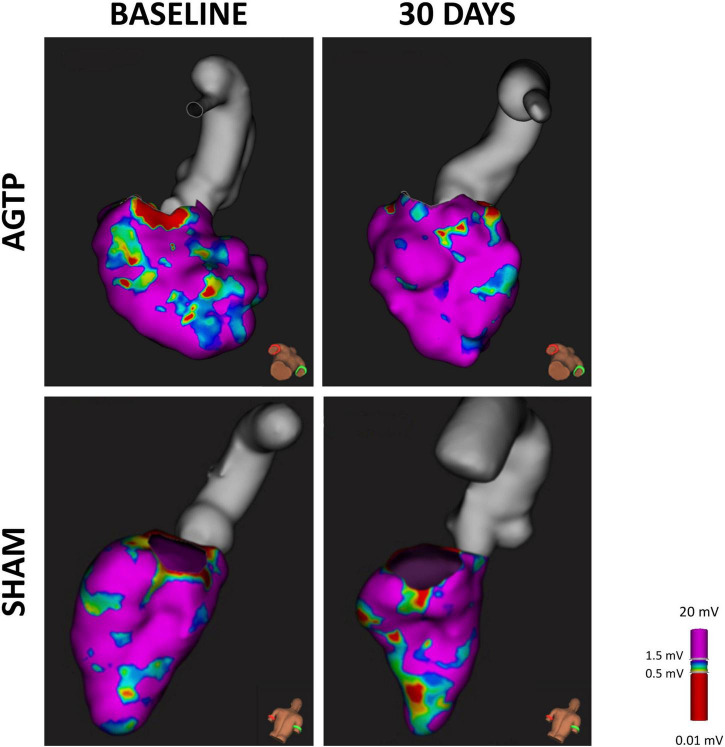
Scar detection by endocardial mapping. High density endocardial mapping of the left ventricle showing post-MI low voltage area. After 30 days, animals receiving AGTP showed a reduction in border zone area (0.5–1 mV) **(upper panel)** whereas those in the Sham group showed overall increase in total scar area **(bottom panel)**.

**FIGURE 5:**
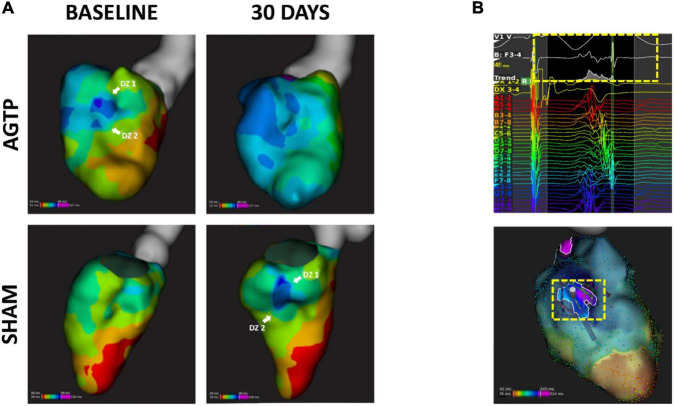
Isochronal mapping and deceleration zones. Representative isochronal activation maps of AGTP **(top)** and sham-surgery **(bottom)** animals; the AGTP group showed significant reduction in the number of deceleration zones over time, whereas an increase was observed in the sham subjects **(A)**. **(B)** Illustrates an **example** of a follow-up high density map of a sham subject exhibiting a late and fractionated electrogram (yellow box) at the latest activation area (infero-basal left ventricular wall).

There was a significant reduction of total scar (–2.2 ± 3.4 cm^2^ vs. + 1.1 ± 0.5 cm^2^, *p* = 0.012) (Table 2) and BZ (–1.9 ± 2.4 cm^2^ vs. + 1.0 ± 1.9 cm^2^, *p* < 0.001) (Figures 3A, 4) areas in AGTP compared to Sham group, with no significant variation of the dense scar area or the unipolar low voltage area (Table 2). The AGTP group showed an improved conduction properties profile compared to Sham group, with a significant reduction in the number of DZ (–0.3 ± 0.9 + 0.8 ± 0.9, *p* = 0.029) (Figures 3B, 5), the area of very-slow (< 0.2 m/s) CV (–3.9 ± 3.8 mm^2^ vs. + 1.6 ± 3.1 mm^2^, *p* = 0.002) (Figure 3C) and the area of highly fractionated electrograms (–1.1 ± 1.3 cm^2^ vs. + 0.6 ± 0.9 cm^2^, *p* = 0.005) (Figure 3D), as well as a trend toward a reduction of the post-QRS activation area (–0.4 ± 1.5 cm^2^ vs. + 0.8 ± 2.8 cm^2^, *p* = 0.069) (Table 2). Intragroup variation analysis is included in Supplementary Table 2.

There were no significant differences in the rate of induction of ventricular arrhythmias in the follow-up EPS between the AGTP and Sham groups [1 (10%) vs. 3 (30%), respectively; *p* = 0.275]. None of the 4 induced ventricular arrhythmias were mapped due to hemodynamic instability (3 VF and 1 fast VT). Intragroup variation analysis is included in Supplementary Table 2.

### Rhythm monitoring

Holter-monitoring was performed in 10 animals (5 of each group). There were no differences between groups in number of PVCs, episodes of non-sustained VT, or total time in VT (Table 2).

### Gene and protein expression

**FIGURE 6:**
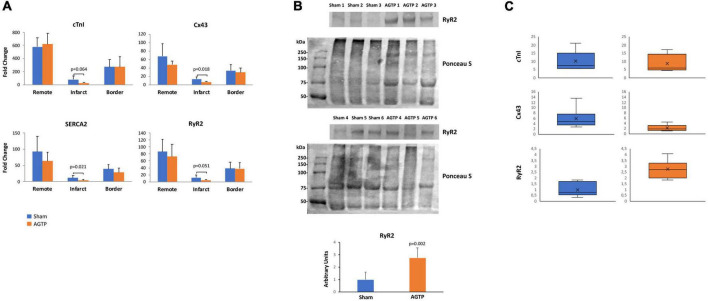
Gene and protein expression profiles. **(A)** *cTnI, Cx43, SERCA2*, and *RyR2* gene expression in sham (*n* = 7) and AGTP-treated (*n* = 6) animals determined by normalizing the expression for each gene to GUSB following the 2^–Δ^ ^Ct^ method. **(B)** Western Blot images of RyR2 presence and the respective Ponceau S staining to depict protein loading **(top)** and the corresponding histogram of RyR2 protein expression of sham and AGTP groups **(bottom)**. **(C)** *cTnI, Cx43*, and *RyR2* protein expression determined by western blot in sham and AGTP-treated animals represented in boxplots.

After MI, the loss of cTnI gene expression in the infarct core region of all pigs was confirmed in AGTP and Sham animals (24.4 ± 10.2 vs. 620.6 ± 167.3 in AGTP, *p* < 0.001; 78.3 ± 55.8 vs. 579.4 ± 140.2 in Sham, *p* < 0.001; respectively), with a trend toward downregulation in AGTP animals (*p* = 0.064), although non-statistically significant. *SERCA2, Cx43*, and *RyR2* genes were decreased in the infarct core of AGTP animals (*p* = 0.021, *p* = 0.018, *p* = 0.051, respectively). Gene expression of *cTnI* was partially retained in the BZ compared to remote myocardium in both the Sham and AGTP groups (53.4 and 56.4%, respectively), confirming the presence of a mixture of viable myocytes and fibrotic scar. In the remote myocardial samples, no significant differences between groups were detected (Figure 6A).

Regarding protein level, western blot analysis in the infarct core showed similar expression of cTnI and Cx43 between Sham (*n* = 6) and AGTP (*n* = 6) groups. However, RyR2 was higher in AGTP group compared to Sham (0.99 ± 0.60 A.U. vs. 2.75 ± 0.81 A.U., respectively; *p* = 0.002) (Figures 6B,C). No differences were detected between groups in terms of SERCA2 and Cx43 protein expression after immunohistochemical analysis. Positive α-SMA myofibroblasts were present in the infarct of all animals suggesting no differences between groups (Supplementary Figure 1, Upper panel). Despite viable cardiomyocytes from the border zone were positive for Cx43 (Supplementary Figure 1, Bottom panel), no differences were detected in infarct core with low-undetectable Cx43 expression. Extended data on western blot analysis is represented in Supplementary Figure 2 and Supplementary Table 3.

## Discussion

The present study showed that cardiac reparative therapy of chronic ischemic scar with AGTP does not increase the risk of spontaneous or induced ventricular arrhythmias and may improve the arrhythmogenic milieu of the scar by reducing the BZ components and the areas of slow conduction, deceleration, and fractionation (Central Illustration).

**Central Illustration:**
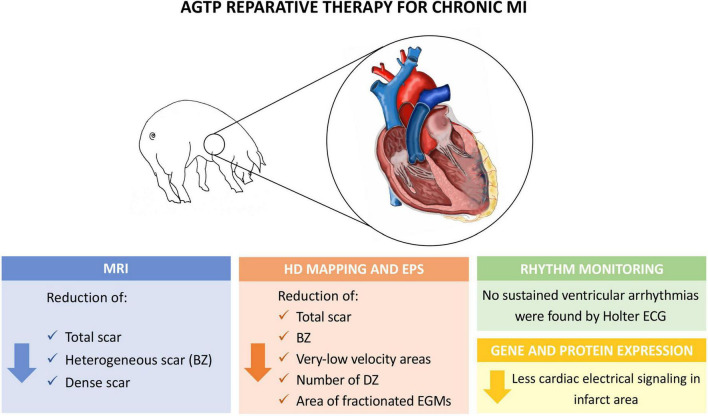
Experimental model and main findings of the study. BZ, border zone; DZ, deceleration zones; EGM, electrogram; EPS, electrophysiological study; HD, high density; MRI, magnetic resonance imaging.

Tissue heterogeneity of chronic ischemic scar is a hallmark of VT substrate (27) and is characterized by a combination of surviving myocardium embedded on diffuse or patchy scar (28). The heterogeneous scar allows for slow, anisotropic conduction (29, 30) through viable myocytes within the scar, historically defined as areas of intermediate bipolar voltage (BZ thresholds 0.5 < 1.5 mV) (22). More recently, advancements in ce-MRI have allowed non-invasive tissue characterization (17–21). Our study showed that AGTP reduces BZ areas of the scar, as assessed by endocardial voltage mapping and by ce-MRI. This effect led to a trend in reducing the number and mass of MRI-detected BZ corridors in the AGTP group, suggesting a potential mechanism for reduced arrhythmic risk.

Our study showed that AGTP may provide additional benefits in terms of arrhythmogenicity by improving the electrophysiological properties of the scar. Treated individuals showed a reduction in the number of DZ and in the size of areas with very-slow CV (<0.2 m/s) and those with a high degree of fractionation. Previous data support that all these conduction abnormalities are commonly present at the critical sites of the VT circuit (26, 31–33). Improvement of overall conduction properties of the ischemic scar could represent an additional mechanistic pathway for reduced risk of ventricular events in individuals receiving reparative therapy with AGTP.

The mechanisms underlying the improvement of CV and the reduction of total scar and BZ in both endocardial HDM and ce-MRI may be multifactorial. First, an increase in vascular supply may have enhanced cell viability by reducing myocardial tissue hypoxia. Previous studies assessing AGTP in chronic MI porcine model showed the presence of micro-vessels connecting the pericardial adipose vascular flap with the underlying ischemic myocardium, which correlated with smaller scars due to increased myocardial salvage after AGTP therapy (4). In acute MI porcine model, cell migration and trafficking from adipose pedicle to the infarcted myocardium was also demonstrated (3). Cardiac adipose tissue contains progenitor cells with inherent cardiac-like phenotype that can differentiate into endothelial cell types (34, 35). Recent data from our group has demonstrated that adipose cardiac tissue-resident progenitor cells may promote local vascularization by secretion of proangiogenic factors (e.g., extracellular vesicles, cytokines) as a paracrine effect (35). Additionally, adipose-derived hormones can exert cardio-protective effects against ischemia by cardiomyocyte apoptosis attenuation, infarct size reduction, and modulation of collagen I/III ratio into the myocardial scar, as we previously reported (3). Consistently with previous data (4), our study showed no benefit in LV contractility in chronic phase of MI. Scar heterogeneity has been shown to be better correlated with arrhythmogenicity than with LV function (36). The lack of improvement in LVEF in AGTP-treated animals is not necessarily related to the electrophysiological remodeling of the scar; thus, AGTP may impact the arrhythmogenic milieu without effect on LV systolic function. Notably, relatively small scars resulted in a remarkable impairment of the LVEF. Unpublished data of another, unrelated set of animals showed the impact of scar location on global LV systolic function. Non-significant differences in LVEF decline were observed at 30-day following anterior (mid LAD, 36.4 ± 7.5%) vs. lateral infarction (first marginal branch of the circumflex artery, 42.5 ± 5.3) (*p* = 0.055), despite significant differences in infarct size (21.1 ± 5 vs. 9.4 ± 6.1% LGE Mass 5SD, respectively; *p* = 0.001). These results suggest that smaller lateral scars may lead to similar impact on global LVEF than more extensive anterior scars.

In addition to the reduced size of scar in AGTP-treated animals, a trend toward a lower cTnI expression might suggest higher homogeneity, although not reaching statistical significance. A lower gene expression of *SERCA2*, *Cx43*, and *RyR2* in the AGTP group may lead to a more homogeneous electrical propagation and contribute to a more unexcitable tissue, which may ultimately reduce arrhythmic events as previously described (37). Specifically, SERCA2 and RyR2 are responsible for Ca2 + intracellular transport (38, 39), and Cx43 for intercellular communication and electric conductance. The downregulation of these proteins in the AGTP group may parallel the reduction of tissue border zone, suggesting a reduction of viable cells intermingled with scar tissue. Cx43 is a component of the gap junction and is involved in the inter-myocyte communication; a lower presence of Cx43 at the myocardial scar and viable cardiomyocytes may contribute to decreased pro-arrhythmic milieu in AGTP-treated animals.

### Study limitations

The number of induced ventricular arrhythmias was low, with no differences between groups. Limited infarct size derived from first marginal artery embolization may have contributed to the low VT inducibility rate and to the high proportion of fast polymorphic VT/VF or poorly tolerated fast monomorphic VTs; thus, no correlation between critical isthmus of the VT and the voltage and activation map could be established. The scar following first marginal occlusion may have eventually affected myocardium beneath the posteromedial papillary muscle; this would likely lead to underestimation of infarct size by endocardial mapping but not by MRI. Epicardial adhesions following prior AGTP/sham surgery preclude epicardial mapping; thus, epicardial arrhythmogenic substrate was not assessed. The study did not include a non-infarcted sham group, according to the 3R principle as it was not expected to add significant information to the study. LV dyssynchrony or regional wall motion abnormalities were not analyzed. Previous data on functional impact of AGTP therapy can be found in previous publications from our group (4). Heart failure related biomarkers (i.e., BNP) were not analyzed. Finally, despite a high SD value of cTnI values, there were no statistically significant differences between groups in post-MI circulant cTnI or in the total scar mass assessed by ce-MRI, therefore ensuring similar baseline MI extension in both groups.

### Clinical perspectives

The AGTP reduces the scar size in chronic MI porcine model (4). In the first-in-human phase I trial (adiFLAP Trial; NCT01473433), no adverse or arrhythmic event related to AGTP were detected (5). In the present study, a thorough assessment of the electrophysiological remodeling of the scar following AGTP was carried out. Our findings suggest that this reparative therapy is safe and may provide protective antiarrhythmic effects, adding a potential benefit against arrhythmic events on top of reducing scar size and heterogeneity and improving LV function. These findings provide additional safety information that will require further confirmation from the ongoing AGTP II randomized clinical trial (phase II-III; NCT02798276) (6). The AGTP is a simple technique that does not require exogenous cell therapy or cardiac tissue engineering products and avoids histocompatibility issues, making the procedure readily available to any patient with chronic MI undergoing cardiac surgery.

## Conclusion

Treatment with AGTP of non-reperfused chronic MI is a safe reparative therapy in terms of arrhythmic risk and provides additional protective effect against adverse electrophysiological remodeling by homogenizing the scar, reducing border zone components, and improving areas of slow conduction and fractionation.

## Data availability statement

The original contributions presented in this study are included in the article/Supplementary material, further inquiries can be directed to the corresponding author/s.

## Ethics statement

The animal study was reviewed and approved by the Animal Experimentation Unit Ethics Committee of the Germans Trias i Pujol Health Research Institute (IGTP) and the Government Authorities (Generalitat de Catalunya; Code: 10558).

## Author contributions

All authors listed have made a substantial, direct, and intellectual contribution to the work, and approved it for publication.

## Footnotes

1
www.paraview.org

## Abbreviations

AGTP: adipose graft transposition procedure
BZ: border zone
Ce-MRI: contrast-enhanced magnetic resonance
CV: conduction velocity
DZ: deceleration zones
EGM: electrograms
EPS: electrophysiological study
HDM: high-density mapping
LV: left ventricle
LVEF: left ventricular ejection fraction
MI: myocardial infarction
PVC: premature ventricular contractions
VF: ventricular fibrillation
VT: ventricular tachycardia.
