# Supplementary material for: Electrophysiological effects of adipose graft transposition procedure (AGTP) on the post-myocardial infarction scar: A multimodal characterization of arrhythmogenic substrate
Source: Front Cardiovasc Med. 2022 Sep 20;9:983001. doi: 10.3389/fcvm.2022.983001 (PMC9530287; doi:10.3389/fcvm.2022.983001)
Supplement: Supplementary file 1 [file Data_Sheet_1.pdf]

Supplementary Material

SUPPLEMENTARY METHODS

Peri-procedural anesthesia

All procedures (myocardial infarction, AGTP and SHAM surgery, and cardiac magnetic resonance) were carried out under general anesthesia and endotracheal intubation. Animals were sedated with an intramuscular (IM) injection of atropine (0.04 mg/kg; Braun, Barcelona, Spain) and pre-anesthetized with dexmedetomidine (0.03 mg/kg, IM; Dexdor®, Orion Pharma, Espoo, Finland), midazolam (0.3 mg/kg, IM; Laboratorios Normon, Barcelona, Spain), and butorphanol (0.3 mg/kg, IM; Butomidor®, Richter Pharma AG, Wels, Austria). Anesthetic induction was performed with an intravenous (IV) bolus of propofol (2 mg/kg; Propovet®, Zoetis, Barcelona, Spain). Animals underwent endotracheal intubation; anesthesia was maintained by 2% isoflurane (IsoVet®, Braun) inhalation. Fentanyl (0.075 mg/kg/45 minutes, IV; Fentadon®, Dechra, Bladel, The Netherlands) was used as intra-operative analgesia(1).

AGTP surgery

After an IV bolus of 1.5 mg/kg atracurium besylate (Sanofi Aventis S.A., Barcelona, Spain), a left lateral thoracotomy in the fourth intercostal space was performed. In all animals, the adipose pericardial tissue was detached from the fibrous layer of the pericardium using dissector scissors and preserving its vascularization. This graft was removed in the sham group; in the AGTP group, the flap was preserved until its transposition. In all animals, the pericardium was excised to expose the lateral wall of the LV and localize the myocardial scar. In AGTP animals, the adipose graft was then transposed to cover the ischemic area and sealed using 0.1–0.2 mL of cyanoacrylic-based surgical glue (co-monomer

of N-butyl-2-cyanoacrylate and metacryloxysulpholane; Glubran®2, Cardiolink, Barcelona, Spain) to the healthy margins of the scar. Animals were then recovered and returned to their facilities(2).

EPS and endocardial mapping

Bandpass filters of 30 to 300 Hz for bipolar signals and 1 to 300 Hz for unipolar signals were applied. During acquisition, local activation time was determined, based on the combination of the bipolar and unipolar electrograms and taking into account the maximum amplitude of the bipolar electrogram and the greatest negative dV/dt of the unipolar electrogram. Both bipolar and unipolar voltage maps were based on the peak-to-peak voltage. A fill threshold of ≤2 mm, as well as limiting interpolation between points to ≤2 mm, was required in all cases. All data points were reviewed and included manually only when a correct contact between the catheter and the endocardium was ensured (near-field EGMs with similar morphology during at least 2 consecutive beats and points within 2 mm from the outermost surface).

Tissue collection

Animals were euthanized at 6-week follow-up by administering sodium thiopental overdose (200 mg/kg). After mid sternotomy, hearts were immediately excised, washed in buffered saline solution, and sliced transversely into three 1-1.5 cm sections (S1-S3) from coronary coil location to the apex. For transcriptomic and proteomic characterization, infarct core, BZ, and remote myocardium biopsies from S1 or S2 sections were immediately collected in Allprotect Tissue Reagent (Qiagen) at room temperature to ensure RNA stabilization of harvested tissue and stored at -80ºC until further use. Additional samples from infarct core were also embedded in OCT and snap-frozen for immunohistochemical analysis.

Gene and protein expression analysis

For gene expression analysis, qRT-PCR reactions (40 cycles) were performed in triplicate using 2 µL of cDNA in a total reaction volume of 10 µL containing 5 µL TaqMan® Fast Advanced Master Mix and 0.5 µL of each porcine FAM-labelled TaqMan® Gene Expression Assay (Applied Biosystems) listed in Supplemental Table 1. Data were collected and analyzed on a LightCycler® 480 RT-PCR system (Roche). Relative quantification was determined by normalizing the expression for each gene to GUSB following the $2^{-\Delta Ct}$ method.

For western blot analysis, 50 µg of protein from the infarct core were electrophorized under reducing (cTnI, CX43, SERCA2, and α- tubulin) and non-reducing conditions (RyR2) on polyacrylamide gels for SDS-PAGE. The samples were electrotransferred to nitrocellulose membranes and incubated with primary antibodies anti-cTnI (1:1000), Cx43 (1:2000), SERCA2 (1:1000) (all three from Abcam), RyR2 (1:1000; Novus Biologicals) and α-tubulin (1:1000; Sigma Aldrich) for 1 hour at RT and with donkey anti-mouse IRDye® 800CW and donkey-anti-rabbit IRDye® 680 secondary antibodies (1:15000; LI-COR Biosciences). The fluorescence intensity (arbitrary units, AU) per band was quantified by densitometry using the Odyssey CLX system and analyzed by Image Studio 2.0 software (LI-COR Biosciences) using the α-tubulin protein as endogenous reference for cTnI, CX43, and SERCA2 quantification, and immunoreactivitiy of RyR2 was normalized to Ponceau S staining (3).

Immunohistochemical analysis

Immunohistochemical analysis was performed in 10-µm snap-frozen sections from infarct core using specific monoclonal antibodies against Connexin 43 (Cx43; 1:200; Abcam), ATPase sarcoplasmic/endoplasmic reticulum Ca2+ transporting 2 (SERCA2; 1:50; Santa Cruz Biotechnology), α-smooth muscle actin (α-SMA; 1:100; Sigma-Aldrich) and cardiac troponin I (cTnI; 1:100; Abcam) (Supplemental figure 1). Cryosections were blocked for 1 hour at RT with 20% horse serum (GIBCO,

Invitrogen, El Prat de Llobregat, Spain), followed by primary antibody incubation (overnight at 4ºC). The antibodies were visualized by incubating the sections with Cy3 (Jackson Immuno Research Labs), Alexa Fluor 488, and Alexa-647 (Molecular probes) (1:500) at RT for 1 h. Slices were finally counterstained for 10 min with 4',6-diamidino-2-phenylindole (1:10.000; Sigma-Aldrich) for nuclei labeling and analyzed using an Axio-Observer Z1 confocal microscope (Zeiss) with Image-Pro Plus software (6.2.1 version; Media Cybernetics, Inc.).

SUPPLEMENTAL REFERENCES

1. Monguió-Tortajada M, Prat-Vidal C, Moron-Font M, et al. Local administration of porcine immunomodulatory, chemotactic and angiogenic extracellular vesicles using engineered cardiac scaffolds for myocardial infarction. Bioact. Mater. 2021;6:3314–3327.

2. Martínez-Falguera D, Fadeuilhe E, Teis A, et al. Myocardial infarction by percutaneous embolization coil deployment in a swine model. J. Vis. Exp. 2021.

3. Sander H, Wallace S, Plouse R, et al. Ponceau S waste: Ponceau S staining for total protein normalization. Anal Biochem. 2019;575:44-53.

SUPPLEMENTAL FIGURES

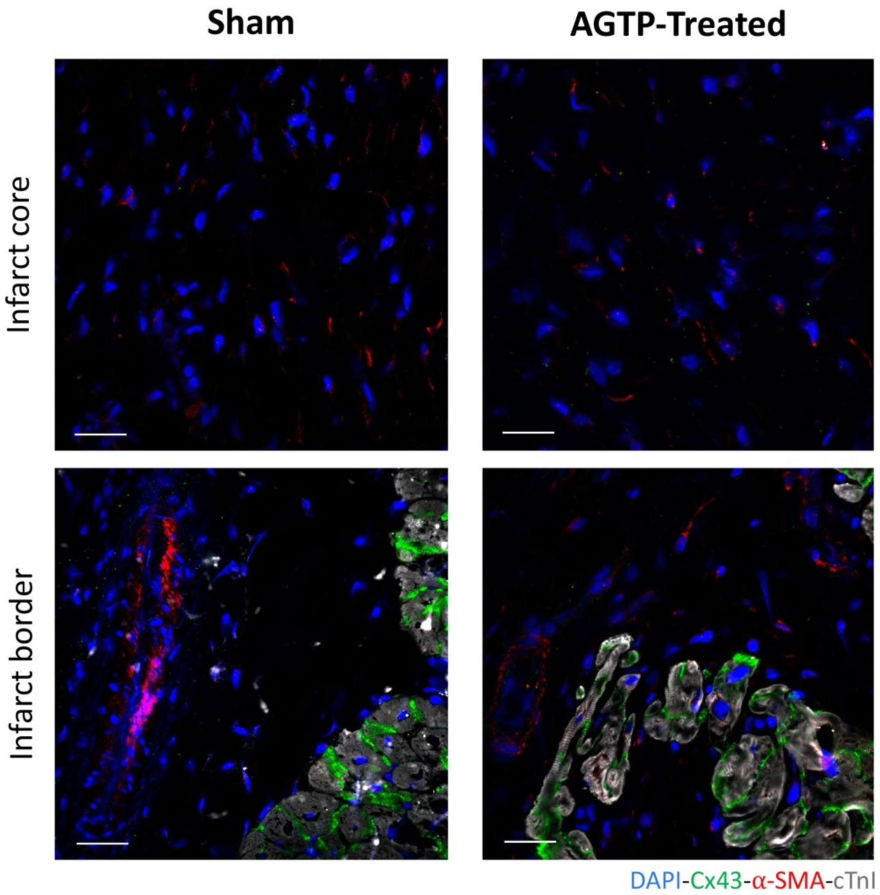

Supplemental Figure 1. Representative immunohistochemical images of Sham and AGTP-treated animals against Connexin 43 (Cx43; green), alpha-Smooth Muscle Actin (α-SMA; red), and cardiac Troponin I (cTnI; white) of infarct core and border zones. Nuclei are counterstained with 4’,6-diamidino-2-phenylindole (DAPI; blue).

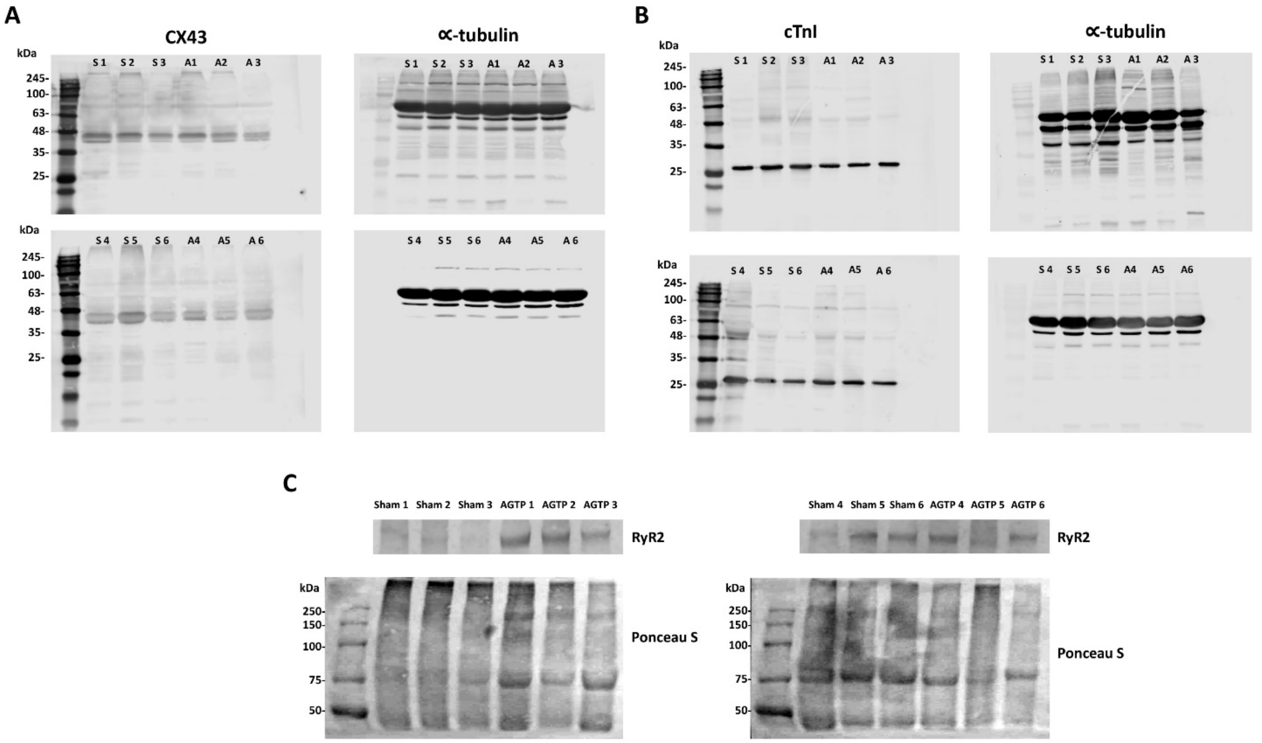

Supplemental Figure 2. Representative images of Western Blot analysis showing all individual bands for Cx43 and α-tubulin (A), cTnI and α-tubulin (B) and RyR2 and Ponceau S (C).

SUPPLEMENTAL TABLES

Supplemental Table 1. TaqMan® Gene Expression Assays used in qRT-PCR analysis.

Gene transcript	NCBI accession number	TaqMan® assay[1]	Amplicon length	Exon boundary
GJA1	NM_001244212	Ss03374839_u1	64	2-2
TNNI3	NM_001098599	Ss03385977_u1	70	7-7
RYR2	XM_021072683	Ss03374536_m1	62	-
SERCA2	NM_213865	Ss03392431_m1	74	16-17
GAPDH	NM_001206359	Ss03375435_u1	75	4-4
PGK1	NM_001099932	Ss03389144_m1	66	4-5
GUSB	NM_001123121	Ss03387751_u1	62	12-12

[1]Thermo Fisher Scientific reference.

Abbreviations: GJA1= gap junction protein alpha 1 (Cx43); TNNI3= troponin I type 3 (cardiac); RYR2= ryanodine receptor 2 (cardiac); SERCA2= ATPase sarcoplasmic/endoplasmic reticulum Ca^{2+} transporting 2; GAPDH= glyceraldehyde-3-phosphate dehydrogenase; PGK1= phosphoglycerate kinase 1; GUSB= glucuronidase beta.

Supplemental Table 2. Intragroup variation of MRI and endocardial HD mapping data.

	Intragroup variation					
	SHAM			AGTP		
	Baseline	30 days post sham Surgery	p	Baseline	30 days post AGTP Surgery	p
iLVEDV (ml, mean±SD)	114±19.2	116.61±17.7	0.693	124.2±17.6	120.9±14.3	0.640
iLVESV (ml, mean±SD)	71.9±21.0	67.7±15.2	0.647	79.9±17.6	74.2±10.3	0.771
LVEF (%, mean±SD)	38.0±11.2	42.4±5.6	0.188	35.6±12.0	38.2±9.6	0.450
Total scar mass (g, mean±SD)	5.8±3.3	7.5±2.8	0.132	8.0±2.9	5.8±1.5	0.039
BZ mass (g, mean±SD)	4.0±2.7	5.1±2.2	0.180	4.9±1.8	3.2±0.8	0.025
Dense scar mass (g, mean±SD)	1.8±1.0	2.3±1.1	0.162	3.1±1.70	2.5±1.3	0.155
Corridors number (g, mean±SD)	0.6±0.7	1±1.2	0.302	1.0±0.8	0.6±0.7	0.442
Corridor mass (g, mean±SD)	0.2±0.3	0.3±0.4	0.653	0.5±0.2	0.3±0.5	0.451
Total scar area (cm^2, mean±SD)	5.4±4.1	6.6±4.1	0.059	8.4±5.2	6.2±3.7	0.070
Border zone area (cm^2, median±IQR)	3.6±2.3	4.8±3.3	0.012	6.2±6.8	3.1±3.4	0.006
Dense scar area (cm^2, mean±SD)	1.7±2.4	1.5±2.0	0.483	1.8±1.7	2.1±1.6	0.527
Unipolar low voltage area (mm^2, median±IQR)	155.3±187.8	117.1±654.6	0.507	80.1±550.5	99.4±456.1	0.575
Area of Velocity <0.2 m/s (mm^2, mean±SD)	1.2±1.8	2.8±2.7	0.123	7.5±8.4	3.6±5.9	0.010
Area of Velocity <0.4 m/s (mm^2, mean±SD)	5.9±9.9	4.9±3.4	0.773	10.0±8.7	5.4±6.5	0.059
Deceleration zones (N, mean±SD)	0.5±0.52	1.2±1.0	0.045	0.9±0.6	0.6±0.5	0.343
Post-QRS activation area (cm^2, median±IQR)	0.7±2.5	1.1±0.9	0.721	1.7±1.2	1.2±2.1	0.006
Area of EGMs with ≥4 deflections (cm^2, mean±SD)	2.8±1.2	3.4±1.5	0.084	4.1±1.9	3.0±2.2	0.032

Abbreviations: EGM= electrogram; iLVEDV= indexed left ventricle end-diastolic volume; iLVESV= indexed left ventricle end-systolic volume; LVEF= left ventricular ejection fraction.

Supplemental Table 3. Western Blot analysis of Cx43, cTnI and RyR2 AGTP and Sham raw data, expressed as arbitrary units.

	cTnI	α-Tubulin	cTnI/ α-Tubulin	Cx43	α-Tubulin	Cx43/ α-Tubulin	RyR2	Ponceau	RyR2/ Ponceau
**Sham1**	**2812857**	**483632.9**	**5.82**	**2004960**	**354582.2**	**5.65**	**66952.1**	**104607.2**	**0.64**
**Sham2**	**3162841**	**395831.3**	**7.99**	**3257322**	**67.1252.3**	**4.85**	**68550.1**	**98531.0**	**0.70**
**Sham3**	**2707833**	**381584**	**7.10**	**1457411**	**5383062**	**2.70**	**30566**	**87203.6**	**0.35**
**Sham4**	**2704640**	**128253..8**	**21.10**	**4605378**	**336375.5**	**13.69**	**94119.5**	**118366.3**	**0.80**
**Sham5**	**1187238**	**213536.8**	**5.60**	**3343840**	**673157.5**	**4.97**	**166207.5**	**99494.1**	**1.67**
**Sham6**	**1514622**	**115845.8**	**13.10**	**1904906**	**515841.3**	**3.69**	**154034.8**	**85272.6**	**1.81**
**AGTP1**	**2199051**	**451391.6**	**4.90**	**1696971**	**630857.1**	**2.69**	**202940.6**	**98898.1**	**2.05**
**AGTP2**	**2523098**	**565326.6**	**4.50**	**971971**	**928745.2**	**1.05**	**199187.6**	**65523.8**	**3.03**
**AGTP3**	**2959628**	**603357.3**	**4.90**	**1815260**	**1187309**	**1.53**	**110880.6**	**59955.0**	**1.84**
**AGTP4**	**2138285**	**124411.8**	**17.19**	**2831271**	**619829.5**	**4.57**	**192633.2**	**64781.2**	**2.97**
**AGTP5**	**2380145**	**172847.8**	**13.77**	**2123401**	**921450.5**	**2.30**	**149464.1**	**60115.8**	**2.49**
**AGTP6**	**1681012**	**231949.7**	**7.25**	**1627971**	**1144269**	**1.42**	**136420.8**	**33283.2**	**4.10**
