# Supplementary figures and images for: Electrophysiological effects of adipose graft transposition procedure (AGTP) on the post-myocardial infarction scar: A multimodal characterization of arrhythmogenic substrate
Source: Front Cardiovasc Med. 2022 Sep 20;9:983001. doi: 10.3389/fcvm.2022.983001 (PMC9530287; doi:10.3389/fcvm.2022.983001)

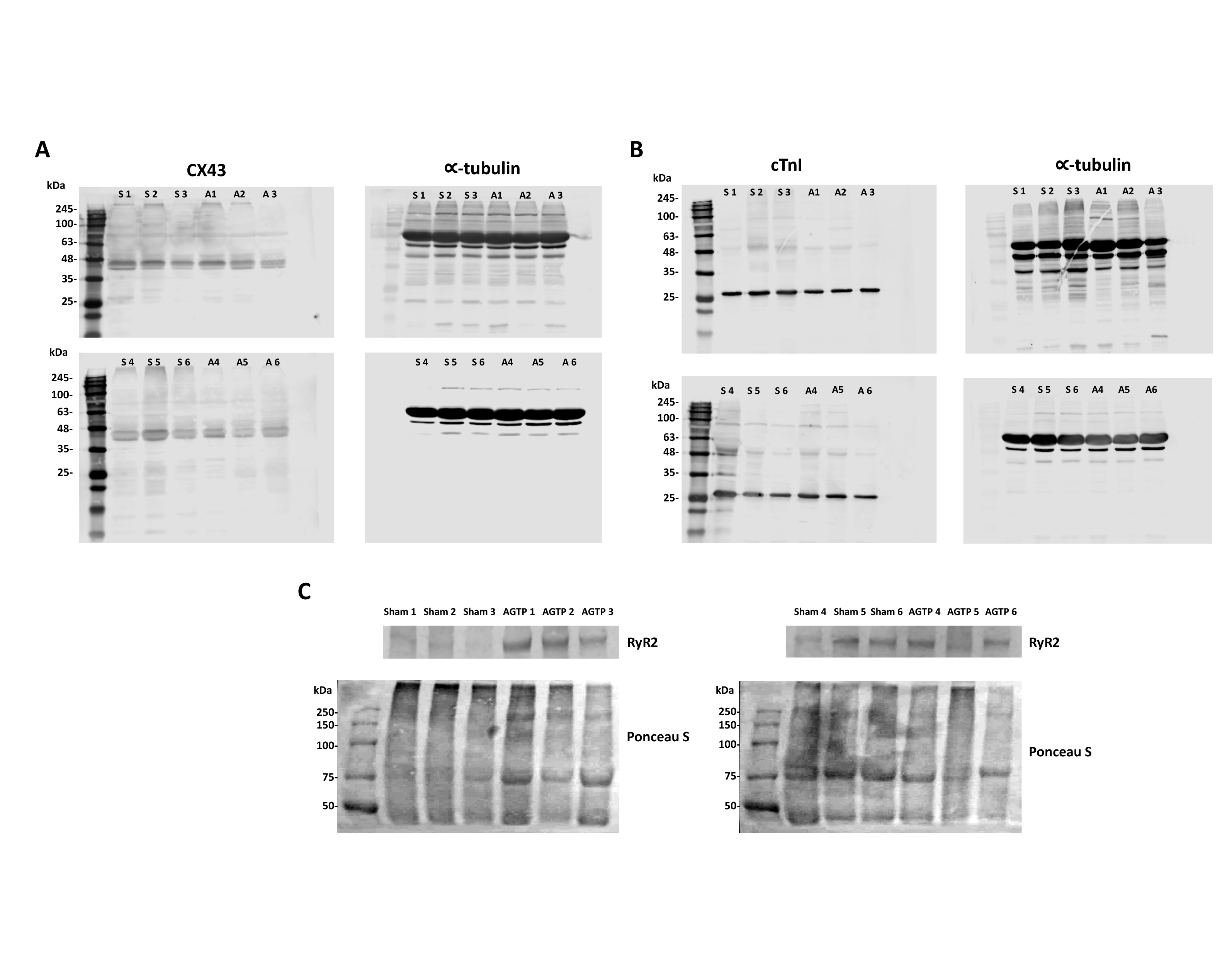

Supplement: Supplementary file 3 [file Image_2.JPEG]
